# The coronavirus pandemic as an analogy for future sustainability challenges

**DOI:** 10.1007/s11625-020-00852-4

**Published:** 2020-08-13

**Authors:** John-Oliver Engler, David J. Abson, Henrik von Wehrden

**Affiliations:** 1 Faculty of Sustainability, Leuphana University of Lüneburg, Lüneburg, Germany; 2 Quantitative Methods of Sustainability Science Group, Universitätsallee 1, 21335 Lüneburg, Germany

**Keywords:** Pandemic, Wicked problem, Sustainability challenge, Behavior change

## Abstract

The current coronavirus outbreak may provide an illustrative analogy for sustainability challenges, exemplifying how challenges such as climate change may become wicked problems demanding novel and drastic solution attempts.

## Introduction

The current coronavirus pandemic is causing an unprecedented worldwide economic and social disruption. The rate of infected people is steadily on the rise (Dong et al. 2020), to a point where the problem has begun to overwhelm regional capacities to respond (2020). It seems that the consequences of this extreme event for the global economy are yet to unfold and hard to predict. In this comment, we argue that the coronavirus crisis resembles, in microcosm and over the short term, the dynamics of global, long-term interrelated sustainability crises—e.g. biodiversity loss, food crisis and climate change—that humanity will face during the twenty-first century. Here we focus specifically on the coronavirus crisis as an analogy for anthropogenic climate change.

## Solving wicked problems

Both the coronavirus crisis and climate change, although occurring on different temporal scales, represent surprisingly similar problem and response dynamics. To this end, the coronavirus pandemic and climate change both feature traits that are characteristic of what has been termed “super wicked problems” (Levin et al. 2012; Rittel and Webber 1973). Specifically, these characteristics are that (1) time is of the essence, (2) those who created the problem are also trying to solve it, (3) there is no or only a weak central authority to address the problem, and (4) actors discount the future irrationally. Such problems defy the standard scientific cascade that builds on a flow of data gathering, analysis, and solution framing (Conklin 2006). Instead, super wicked problems demand a problem and solution-oriented approach that is continuous in action and generally based around a suite of concurrent interventions, rather than a single pre-defined solution. Furthermore, wicked problems are characterized by a complexity that hampers transferability based on previous experience, and leave little room for repeated attempts to reach a solution. In the following four sections, we highlight characteristics that unify the coronavirus pandemic and climate change, and outline how to evaluate and approach solutions for these challenges.

## Finding solution sets

The coronavirus crisis is threatening to rapidly overwhelm the capacities of health care systems of severely affected countries around the globe (2020). Disruption or even collapse of the health care systems and the associated responses are likely to lead to long-term impacts affecting the economy, civil institutions, and governments. The economies of the most severely affected countries may be afflicted for years if not decades, yet the most drastic impact is clearly local and in the immediate future. Containment strategies that aim at tracking the coronavirus outbreak from single patients have largely failed, also because some political leaders decried the epidemic as a ‘hoax’. As a result, the epidemic has become a pandemic that features a complex and gradual increase of infections across both time and space. Governments have switched to mitigation and adaptation strategies. The ecological and socio-economic impacts of climate change are similarly complex and gradually increasing across both space and time. Containment would have been possible, but it is now too late. Given this complexity there is a need for a systemic understanding of the principle drivers of the problems, rather than attribution of causal factors to single events. Hence, both climate change and coronavirus pandemic will require a set of coherent and coordinated solutions that address both the immediate problems and the underlying causes of the crisis.

## Novel and bold solutions are needed

It is becoming evident that the world community was ill prepared for the coronavirus pandemic, although experts flagged the problem early on (World Health Organization 2020). Similarly, knowledge about the impact of climate change is widely available [e.g. (Field et al. 2014), https://www.nature.com/nclimate]. Still, many experts agree that to date the global reaction to climate change has been insufficient and policy proposals would need to better account for the effect of culture as a mediator in societal reactions to climate change (Adger et al. 2013). As with the coronavirus pandemic, reactions to the threats of climate change have to be bold and novel (Rapley and Meyer 2014). In particular, global leaders need to cooperate with each other rather than to defect. For both climate change and the coronavirus crisis interventions must be attempted, despite a current lack of full knowledge of the outcome or effectiveness of these interventions. With both coronavirus and climate change interventions based on existing structures and institutions have been apparently inadequate, indicating a demand for novel interventions, including the creation of new institutions and radical changes in governance and lifestyles.

## Problem escalation results in non-scalable, time sensitive solution sets

Responses to the coronavirus crisis demand urgent measures to minimize further spread, and despite unprecedented global measures already in place, halting the outbreak’s momentum is challenging. As with the coronavirus crisis, urgent actions to tackle anthropogenic climate change are now required as the impacts of changing climate are increasing in both scale and pace (Pittock 2013). The future adverse effects of climate change are likely to accelerate, with the costs of adaptation measures increasing over time (Stern 2008). Moreover, as with coronavirus, if not tackled in a timely manner, climate change is likely to disrupt the social systems that are the basis for both mitigation and adaptation. Therefore, a rapid and early response to climate change is crucial, and there will likely be no second attempt to solve the problem. Moreover, the solution sets become increasingly more challenging to implement and more disruptive to the system as the multiple manifestations of the crises increase. In other words, the solution sets are not fixed and not ‘scalable’, but rather constantly changing in relation to the escalating problem.

## Behavior change as a key approach

Both challenges demand solution-oriented action with multiple interventions that address the systemic causes of the crises. Furthermore, the changing nature of the crises means that a single specific solution may not be possible and interventions need to be continually adjusted and reassessed to changing conditions. Behavior change represents a crucial component in addressing both crises [(Fischer 2012; Engler et al. 2019)]. Arresting the spread of coronavirus requires challenging deeply held societal behaviors values such as the freedom of movement and travel. Similarly, tackling climate change requires us to address our ingrained behaviors related to resource consumption. While the coronavirus crisis unfolds on a short to mid-term scale, the impacts of climate change are starting to manifest themselves only now. The crisis caused by the coronavirus pandemic may thus foreshadow the disruptive force with which other wicked problems such as anthropogenic climate change might affect our global economic system and society. What unites these problems is the fact that the time to act is now.
